# The Gac-Rsm and SadB Signal Transduction Pathways Converge on AlgU to Downregulate Motility in *Pseudomonas fluorescens*

**DOI:** 10.1371/journal.pone.0031765

**Published:** 2012-02-20

**Authors:** Francisco Martínez-Granero, Ana Navazo, Emma Barahona, Miguel Redondo-Nieto, Rafael Rivilla, Marta Martín

**Affiliations:** Departamento de Biología, Universidad Autónoma de Madrid, Madrid, Spain; Vrije Universiteit Brussel, Belgium

## Abstract

Flagella mediated motility in *Pseudomonas fluorescens* F113 is tightly regulated. We have previously shown that motility is repressed by the GacA/GacS system and by SadB through downregulation of the *fleQ* gene, encoding the master regulator of the synthesis of flagellar components, including the flagellin FliC. Here we show that both regulatory pathways converge in the regulation of transcription and possibly translation of the *algU* gene, which encodes a sigma factor. AlgU is required for multiple functions, including the expression of the *amrZ* gene which encodes a transcriptional repressor of *fleQ*. Gac regulation of *algU* occurs during exponential growth and is exerted through the RNA binding proteins RsmA and RsmE but not RsmI. RNA immunoprecipitation assays have shown that the RsmA protein binds to a polycistronic mRNA encoding *algU*, *mucA*, *mucB* and *mucD*, resulting in lower levels of *algU*. We propose a model for repression of the synthesis of the flagellar apparatus linking extracellular and intracellular signalling with the levels of AlgU and a new physiological role for the Gac system in the downregulation of flagella biosynthesis during exponential growth.

## Introduction

The Gac system (GacA/GacS) conforms a conserved [1] global regulatory system that regulates the production of the majority of exoproducts and virulence factors in the pseudomonads, independently of their life-style [2]–[6]. In the opportunistic pathogen *Pseudomonas aeruginosa*, the Gac system positively regulates the production of the autoinducer N-butyryl-homoserine lactone and the formation of the virulence factors pyocyanin, cyanide, lipase [7] and elastase [8], being necessary for full virulence in animal and plant hosts [9]. The Gac system also regulates most of the virulence factors that have been identified in the insect pathogen *P. entomophila* [10]. In phytopathogenic pseudomonads, such as *P. syringae*, the Gac system has been implicated in lesion formation, production of protease and the phytotoxin syringomycin [11], swarming motility [12] and alginate production [13], acting as a master regulator [14]. In saprophytic pseudomonads such as *P. fluorescens*, *P. putida*, *P. aureofaciens*, and others, the Gac system has been shown to regulate the production of secondary metabolites such as the fungicide 2,4-diacetylphloroglucinol (DAPG), cyanide, pyoluteorin, phenazine, the phytohormone indole-3-acetic acid [15]–[18], extracellular enzymes and fluorescent siderophores [19], [20], and lipopeptides such as amphisin [21] and putisolvin [22]. Mutations in the Gac system often result in the loss of biocontrol ability [17], [23].

The Gac system acts as an activator, in the regulation of the production of most of these exoproducts. This system, in response to a yet unidentified signal produced during the transition to stationary phase [24], activates the transcription of several small regulatory RNAs termed *rsmX*, *rsmY* and *rsmZ* [25], [26]. Different *Pseudomonas* produce one, two or three of these sRNAs [15], [25], [27]. In turn, the small RNAs titrate RNA-binding proteins (RsmA, RsmE and in some strains RsmI) that in the absence of the small RNAs bind to the 5′ regions of target messenger RNAs repressing their translation [28], [29]. However, in a few cases, negative regulation by the Gac system has been observed. This is the case for rhamnolipids and lipase production, and swarming motility in *P. aeruginosa* PAO1 [30].

We have previously shown that swimming motility of *Pseudomonas fluorescens* F113 which is important for rhizosphere colonization and biocontrol ability is also under negative control by the Gac system, since mutants affected in either of the *gac* genes produce larger swimming haloes than the wild-type strain [31], [32].We have also shown that this downregulation occurs through the repression of the flagellar master regulatory gene *fleQ*, resulting in reduced production of proteins of the flagellar apparatus, including the flagellin FliC [33].

The *sadB* gene encodes a cytoplasmic signal transduction protein that was initially characterized as a protein implicated in surface attachment in the initial steps of biofilm formation [34] and in repressing swarming motility by rhamnolipid sensing [35]. This protein contains a modified HD(N)-GYP domain although no phosphodiesterase activity has been demonstrated [35]. SadB has also been implicated in downregulation of swimming motility in F113, and this regulation is also mediated by downregulation of *fleQ* [33]. The aim of this work was to investigate the mechanism of negative regulation of swimming motility by the Gac system and SadB, and how they converge in the repression of *fleQ*.

## Results

### The Gac system regulates motility through the Rsm pathway

**Figure 1:**
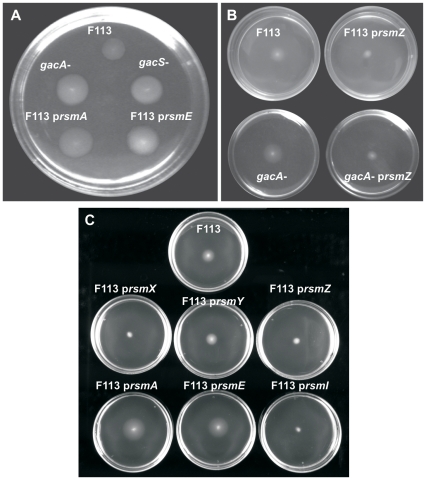
The Gac system regulates motility through the Rsm pathway. (**A**) Analysis of the swimming motility of *P. fluorescens* F113 wild-type, F113 *gacA* mutant, F113 *gacS* mutant, F113 p*rsmA*, and F113 p*rsmE*. (**B**) Swimming motility of F113 wild-type strain and its isogenic *gacA* mutant harbouring the empty vector pVLT31 or pVLT31-*rsmZ* (p*rsmZ*). (**C**) Swimming motility of F113 wild-type strain harbouring the empty vector pVLT31, pVLT31-*rsmX* (p*rsmX*), pVLT31-*rsmY* (p*rsmY*), pVLT31-*rsmZ* (p*rsmZ*), pVLT31-*rsmA* (p*rsmA*), pVLT31-*rsmE* (p*rsmE*) or pVLT31-*rsmI* (p*rsmI*).

Sequence BLAST analysis of the genomic sequence of *P. fluorescens* F113 [36] showed that this bacterium possess genes encoding three Rsm proteins (*rsmA,E* and *I*) and three small RNAs (*rsmX*, *Y* and *Z*) homologous to their counterparts in other pseudomonads. Since Rsm proteins and *rsm* sRNAs have been shown to be in most cases redundant, we have chosen to analyze strains overexpresing *rsmA*, *rsmE*, *rsmI* and *rsmX*, *Y and Z*. To test whether negative regulation of motility occurred through the Rsm pathway, we hypothesized that in this case the overproduction of either of the Rsm proteins would mimic the phenotype of a *gac* mutant. In order to overexpress the *P. fluorescens* F113 *rsmA*, *rsmE* and *rsmI* genes, the amplified genes were cloned into vector pVLT31 (Table S1), under the control of the *Ptac* promoter and introduced into *P. fluorescens* F113 by triparental mating, to generate strains F113 p*rsmA*, F113 p*rsmE* and F113 p*rsmI*. As shown in Fig. 1A and 1C, while the wild-type strain F113 showed normal motility, overexpression of either of the *rsmA* and *E* genes in F113 resulted in enhanced motility, a phenotype identical to the *gacA* and *gacS* mutants. However, overexpression of *rsmI* did not result in an increase in swimming motility, but in a slight decrease. Plasmid overexpression of the *rsmZ* and *rsmX* sRNA under the control of the same promoter (p*rsmZ* and p*rsmX*) resulted in a reduction of motility in the wild-type strain and suppressed the swimming phenotype of a *gacA* mutant (Fig. 1B–1C). Conversely, overexpression of *rsmY* did not have an effect on swimming motility. These results confirm that the Gac and Rsm systems act in the same pathway in repressing motility in *P. fluorescens*, although *rsmI* and *Y* do not participate in this regulation.

### Negative regulation of motility by the Gac system acts through downregulation of the *fleQ* gene transcription during exponential phase

**Figure 2:**
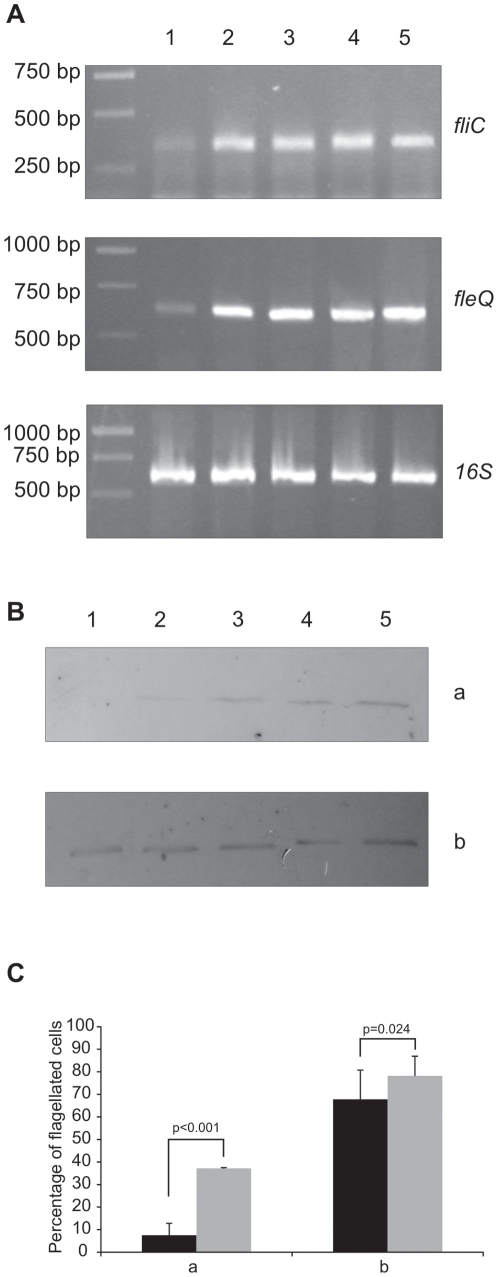
Negative regulation of motility by the Gac system acts through downregulation of the *fleQ* gene transcription during exponential phase. (**A**) RT-PCR expression analysis of *fliC* (primers fliCF-R), *fleQ* (primers fleQF-R), and *16S* (primers 16SF-R) genes of F113 (1), *gacA* ***^−^*** (2), *gacS* ***^−^*** (3), F113 p*rsmA* (4), and F113 p*rsmE* (5). (**B**) Western blot analysis of external proteins from F113 (1), *gacA* ***^−^*** (2), *gacS* ***^−^*** (3), F113 p*rsmA* (4), and F113 p*rsmE* (5) during exponential phase (O.D._600_ = 0.3) (a), and stationary phase (O.D._600_ = 3.5) (b), reacted with an anti-flagellin antiserum. The observed band is approximately 35 KDa and corresponds to FliC. (**C**) Percentage of flagellated cells of F113 wild-type (black bar) or *gacS* ***^−^*** (grey bar) during exponential phase (O.D._600_ = 0.3) (a), and stationary phase (O.D._600_ = 3.5) (b). Statistical significance is shown.

The *fleQ* gene encodes the major regulator of flagellar biosynthesis [37], [38] in pseudomonads. We have previously shown that hypermotile phenotypic variants of *P. fluorescens* F113 were characterized by overproduction of flagellin (FliC) and longer flagella [39]. Furthermore, we have shown that the GacAS pathway downregulates motility through repression of *fleQ* expression [33]. The expression of *fleQ* and *fliC* genes was also higher in the strains that overexpressed the *rsmA* and *rsmE* genes (Fig. 2A). These results clearly show that the negative regulation of motility by the Gac system acts through the Rsm pathway on the flagellar filament synthesis, by repressing the expression of the *fleQ* gene, resulting on a lower level of expression of genes encoding structural elements of the flagellum, including the *fliC* gene, which encodes flagellin.

Since the Gac system regulates secondary metabolism, especially at the transition from exponential to stationary growth, we hypothesized that the role of the Gac system on motility could be to downregulate flagellar synthesis during exponential growth. To test this hypothesis, total extracellular proteins from the wild-type strain, both *gac* mutants and the strains overexpressing the *rsmA*/*E* genes were precipitated from the growth medium during exponential phase (O.D._600_ = 0.3) and late stationary phase (O.D._600_ = 3.5). These proteins were probed with an anti-FliC (flagellin) antiserum [40]. As shown in Fig. 2B, during exponential phase the *gac* mutants and the strains overexpressing either of the *rsm* genes produced a higher amount of flagellin than the wild-type strain. However, during late stationary phase no differences in flagellin production were observed with the wild-type strain. Furthermore, transmission electron microscopy of negatively stained samples from the *gac* mutants and the wild-type strain showed that the percentage of flagellated cells were higher in the *gacS* mutant than in the wild-type strain during exponential growth (8% for wild-type strain, and 37% for *gacS* mutant) but not during stationary phase (68% for wild-type strain, and 78% for *gacS* mutant) (Fig. 2C). These results support the hypothesis of the role of the Gac system limiting flagella biosynthesis during exponential growth phase.

### Gac-mediated downregulation of *fleQ* expression is independent of Vfr but dependent on AmrZ and AlgU

**Figure 3:**
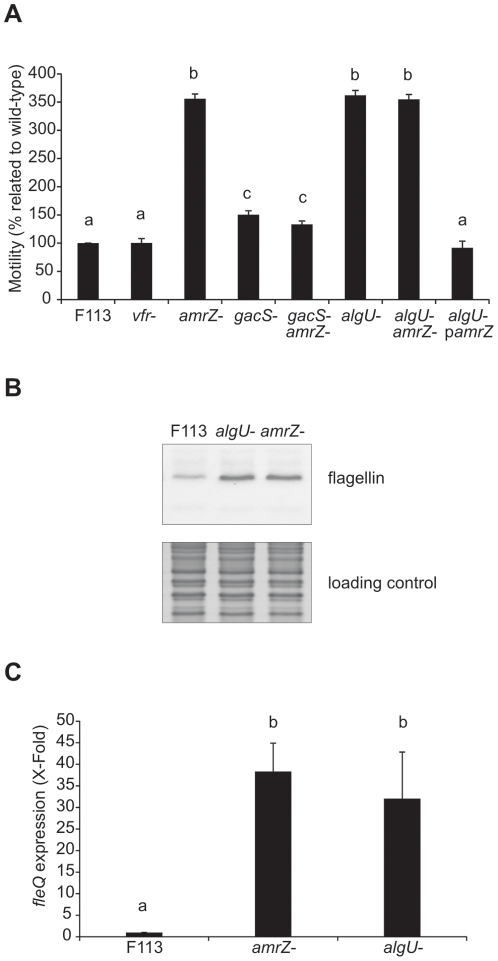
Gac-mediated downregulation of *fleQ* expression is independent of Vfr but dependent on AmrZ and AlgU. (**A**) Analysis of the swimming motility of F113 wild-type, mutants in the Gac-AlgU cascade, and complemented *amrZ* (p*amrZ*). Different letters indicate significant statistical difference (p<0.05). At 18 h, F113 wild-type strain swimming halo diameter is 11±0.55 (mm). (**B**) Western blot analysis of extracellular proteins from F113 wild-type strain and its isogenic mutants *amrZ* ***^−^*** and *algU* ***^−^***, reacted with an anti-flagellin antiserum. Loading control corresponds to a Coomassie-stained gel portion. (**C**) qRT-PCR expression analysis of the *fleQ* gene (primers qfleQF-R) in F113 wild-type, *amrZ* ***^−^*** and *algU* ***^−^***. *16S* gene expression (primers 16SF-R) was used for normalization. To control for DNA contamination, PCR of RNA was performed using the same primer pairs. Different letters indicate significant statistical differences (p<0.05).

Gac regulation through the Rsm pathway takes place at the translational level since the RsmA and E proteins bind specific messenger RNAs blocking their translation [41], [42]. For negative regulation of motility, the RNA blocked should encode a repressor of *fleQ* transcription. Although several proteins such as MorA, FleN and AlgU have been shown to modulate *fleQ* expression in different pseudomonads [43]–[45], a direct role in repressing *fleQ* transcription by binding to the promoter region has been suggested for the global regulatory protein Vfr [46] and for AmrZ [47] in *P. aeruginosa*. Furthermore, Vfr has been implicated in the regulation of two Gac-controlled traits in *P. aeruginosa*: elastase and pyocyanin production [48]. Since the F113 *fleQ* promoter region contains a putative Vfr binding site, we decided to test whether Vfr was implicated in Gac-mediated *fleQ* downregulation. For this purpose, we used primers vfrF and vfrR (Table S2) to amplify an internal fragment of the *vfr* gene from F113 genomic DNA and cloned it into pVIK107 (Table S1). This construct was integrated into the F113 genome by homologous recombination and the resulting strain F113 *vfr* ***^−^*** was checked by PCR and Southern-blot, using the amplified *vfr* fragment as the probe. However, the *vfr* mutant did not show significant differences in motility compared to the wild-type strain (Fig. 3A), indicating that Vfr is not implicated in this pathway. In order to generate an *amrZ* mutant, an internal fragment of the gene was amplified with primers amrZF and amrZR (Table S2) and cloned into the pK19*mobsacB* vector (Table S1). The resulting plasmid was integrated into F113 genome by homologous recombination and the disruption of the gene was checked by PCR and Southern blot. As shown in Fig. 3A the *amrZ* mutant showed enhanced motility with respect to the wild-type strain and the *gac* mutants. Expression of *fleQ* and *fliC* was higher in the *amrZ* mutant than in the wild-type (Fig. 3B–3C). In order to test whether the Gac system and AmrZ were acting in the same pathway, a double mutant *gacS-amrZ* was constructed by disruption of the *gacS* gene in an *amrZ* mutant background (Table S1). The resulting double mutant had the same motility phenotype than the *gacS* mutant (Fig. 3A) showing genetic interaction, indicating that both genes participate in the same regulatory pathway. Since AlgU has also been implicated in regulation of motility in other pseudomonads, and it has been described as the sigma factor required for the expression of *amrZ* [47], we constructed an *algU* mutant by gene disruption by cloning an internal fragment of the *algU* gene into pK19*mobsacB* vector (Table S1). This plasmid was integrated into the F113 genome by homologous recombination and the disruption of *algU* was checked by PCR and Southern blot. The *algU* mutant showed a similar swimming phenotype than the *amrZ* mutant (Fig. 3A) and overexpressed *fleQ* and *fliC* (Fig. 3B–3C). A double mutant *algU-amrZ* showed the same phenotype than the independent mutants (Fig. 3A), indicating that both genes act in the same signalling pathway. Furthermore, ectopic expression of *amrZ* (p*amrZ*) in an *algU* mutant background, restored wild-type motility (Fig. 3A), showing that the phenotype of the *algU* mutant is caused by the lack of expression of *amrZ*.

### RsmA binds the *algUmucABD* polycistronic mRNA

**Figure 4:**
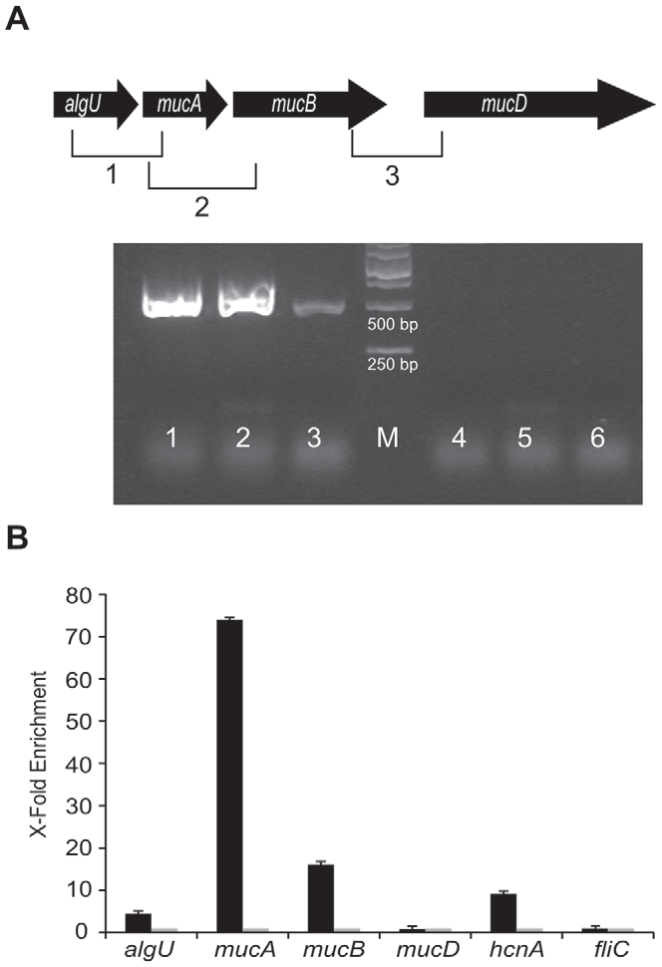
RsmA binds the *algUmucABD* polycistronic mRNA. (**A**) RT-PCR of adjacent genes in the polycistronic mRNA *algU*-*mucA*-*mucB*-*mucD*. PCR of cDNA using the primer pairs qalgUF-qmucAR (lane 1), qmucAF-qmucBR (lane 2) and qmucBF2-qmucDR (lane 3) or PCR of RNA using the same primer pairs qalgUF-mucAR (lane 4), qmucAF-qmucBR (lane 5) and qmucBF2-qmucDR (lane 6), M marker. (**B**) RNA-IP assay of F113 wild-type strain harbouring the pVLT31-rsmAHA plasmid. qRT-PCR of HA-immunoprecipitated RNA (black bar) or IgG-immunoprecipitated RNA (mock, grey bar) using the primer pairs qalgUF-R (*algU*), qmucAF-R (*mucA*), qmucBF-R (*mucB*), qmucDF-R (*mucD*), qhcnAF-R (*hcnA*) and qfliCF-R (*fliC*). The *fliC* gene was used for normalization.

In *P fluorescens* F113 the *algU* gene is followed by three genes encoding the antisigma factors MucA and B and the protease MucD. In order to test whether these four genes form an operon, an RT-PCR experiment was performed by designing primers for the co-amplification of adjacent genes. As shown in Fig. 4A, amplicons of the expected size were obtained for *algU-mucA*, *mucA-mucB* and *mucB-mucD* indicating that the three genes are encoded in a polycistronic mRNA. In order to test the hypothesis of RsmA binding to this mRNA, an RNA immunoprecipitation assay was performed. For this assay a C-Terminus HA-tagged RsmA protein was generated by PCR and cloned into expression vector pVLT31 (Table S1). This RsmA-HA protein was functional, since overexpression of the construct mimics the phenotype of the overexpression of the *rsmA* gene (not shown). RNA immunoprecipitation showed that the RsmA protein binds to the *algUmucABD* mRNA (Fig. 4B). Furthermore, binding of RsmA to this RNA was stronger than binding to the *hcnA* mRNA, that has been previously shown to be post-transcriptionally regulated by binding of RsmA/RsmE to its 5′ region [28], [29]. Binding of RsmA seems to be located in the region upstream of *mucA*, since the higher amount of immunoprecipitated RNA is located in this region.

### SadB and GacAS regulate *algU* expression

**Figure 5:**
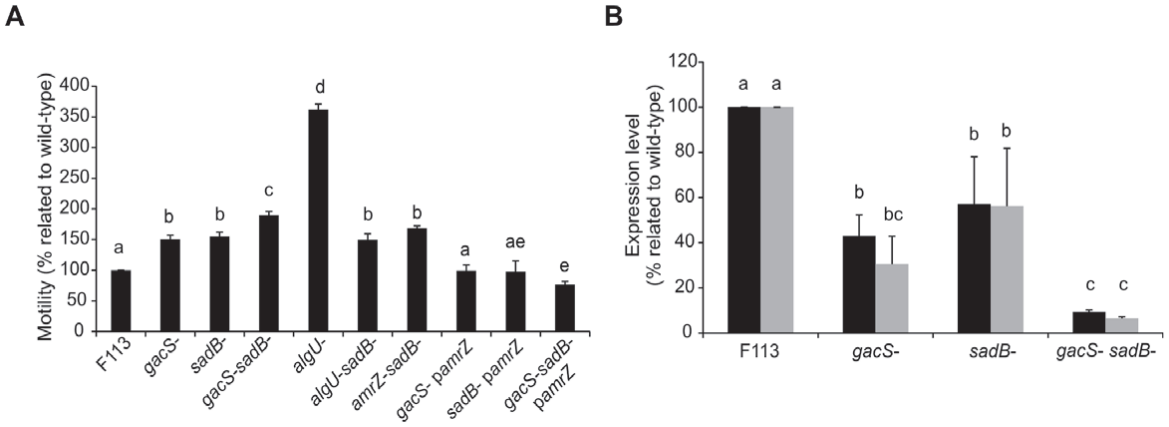
SadB and GacAS regulate *algU* expression. (**A**) Analysis of the swimming motility of F113 wild-type, mutants in the Gac-SadB-AlgU cascade, and *amrZ* complementation (p*amrZ*). Different letters indicate significant statistical differences (p<0.05). At 18 h, F113 wild-type strain swimming halo diameter is 11±0.55 (mm). (**B**) qRT-PCR expression analysis of *algU* (black) and *amrZ* (grey) genes (primers qalgUF-R and qamrZF-R, respectively) in F113 wild-type, *gacS* ***^−^***, *sadB* ***^−^***, and double mutant *gacS-sadB*. *16S* gene expression (primers 16SF-R) was used for normalization. Different letters indicate significant statistical differences (p<0.05).

We have previously shown that not only GacAS but also SadB downregulates motility through FleQ [33]. In order to test whether *fleQ* regulation by SadB was through the AlgU-AmrZ pathway, double mutants *algU-sadB* and *amrZ-sadB* were constructed. As shown in Fig. 5A, both double mutants presented the same phenotype than the *sadB* mutant, indicating that the three genes act in the same pathway. We have previously shown [33] that a *sadB-gacS* mutant presents an additive swimming phenotype, indicating that the Gac and SadB pathways converge on the regulation of *algU*. We also analyzed the expression of the *algU* and *amrZ* in the *gacS*, *sadB* and *gacS-sadB* mutant backgrounds. Expression of both genes was clearly reduced in the individual mutants and very low in the double mutant (Fig. 5B), confirming the cooperative regulation of *algU* by the Gac and SadB pathways. Furthermore, ectopic expression of the *amrZ* gene (p*amrZ*) in the *gacS*, *sadB* and *gacS-sadB* mutants complemented the swimming phenotype of the mutants (Fig. 5A).

## Discussion

Although for most traits the Gac system acts as a positive regulator, for some traits such as swarming motility and rhamnolipids and lipase production it may function as a negative regulator [30]. This negative role of the Gac system is especially clear for swimming motility in *P. fluorescens* F113, since mutations in the *gacA* or *gacS* genes results in increased motility [31], [32]. The relevance of this trait and of the Gac system for rhizosphere colonization is highlighted by the fact that phenotypic variants arising during rhizosphere colonization harbour mutations in the *gac* genes, being more motile than the wild-type strain. Furthermore, several of these variants, selected because of their increased motility, were more competitive for rhizosphere colonization than the wild-type strain [32]. The finding that the FliC and FliD proteins are among the most highly overproduced proteins in *gac* mutants, in *P. aeruginosa* [27] and *P. fluorescens* [3], suggests that negative regulation of motility by the Gac system may be a general feature in pseudomonads.

Activation through the Gac system occurs post-transcriptionally. Briefly, an unidentified signal stimulates autophosphorylation of the GacS sensor [24]. The phosphate group is then transferred to the response regulator GacA by a phospho-relay mechanism, activating directly or indirectly the transcription of genes encoding small RNAs, termed *rsmX*, *Y* and *Z* [49]. These riboregulators bind to RNA-binding proteins such as RsmA, E and I, which have the ability to bind specific mRNAs blocking their translation [28], [50]. In such system, an active Gac system results in the Rsm proteins bound to the small regulatory RNAs and therefore the target mRNAs are translated. Conversely, in the absence of a functional Gac system (for instance strains harbouring a *gac* mutation), the Rsm proteins would be bound to their target mRNAs that would not be translated [51]. This model easily explains positive regulation, since translation of the target mRNAs is required for the production of the trait. Here we show that the Rsm pathway (excluding *rsmI* and *Y*) is also used for negative regulation of motility in *P. fluorescens*, since overexpression of either of the *rsmA* or *E* genes mimics the phenotypes of the *gac* mutants. Our results also show that for repression of swimming motility, the RsmA and RsmE proteins are functionally equivalent (Fig. 1A). This functional equivalence has also been shown for other positively regulated traits such as exoprotease, hydrogen cyanide, and 2,4-diacetylphloroglucinol in *P. fluorescens* CHAO [42]. However, it is not true for all Rsm-controlled traits. We have shown here that neither *rsmI* nor *rsmY* participate in negative regulation of motility in strain F113. It has also been shown that in *P. aeruginosa*, the BfiSR two-component system regulates biofilm formation through *rsmZ* but not through *rsmY* [52]. It is interesting to note that several pseudomonads, such as *P. aeruginosa*, produce a single Rsm protein [53] whereas other as it is the case for *P. fluorescens* F113, produce more than two Rsm-like proteins [42].

We have previously shown that hypermotile phenotypic variants of strain F113 isolated from the rhizosphere harboured *gac* mutations, produced higher amounts of the FliC protein and possessed longer flagella than the wild-type strain [39]. Since the major activator of flagellar synthesis is the FleQ protein [38], we decided to test whether the Gac system acted through the *fleQ* gene to regulate swimming motility. Our results clearly show that the Gac system dramatically influences the level of transcription of the *fleQ* and *fliC* genes and that this influence is enforced through the RsmA and RsmE proteins (Fig. 2A). These results are consistent with those reported in *P. aeruginosa* that showed that in a *gacA* and *rsmYZ* mutants, FliC and FliD (the flagellar cap protein) had increased expression (between 7.5 and 10.2-fold) when compared to wild-type strain, being the most overproduced proteins in both mutants [27].

Since Gac regulation of motility occurs through the Rsm pathway, a direct effect on the transcription of activators such as *fleQ* can be discarded. Two alternative ways are possible. The RsmA and E proteins could bind to the mRNA of the transcriptional activators stabilizing them or the Rsm proteins would bind to the mRNAs encoding transcriptional repressors of the activator genes. The former possibility has been shown to occur with the RsmA homologue CsrA in *Escherichia coli* [54]. In this bacterium, CsrA binds the mRNA of the *flhDC* genes, which encode the master operon regulating flagellar biosynthesis. The second possibility, i.e. the RsmA and E binding of a mRNAs encoding a transcriptional repressor of *fleQ* could explain the observed phenotype of *gac* mutants. We have tested two putative repressors, Vfr and AmrZ [46], [47]. Our results discard the implication of Vfr in Gac regulation of swimming motility (Fig. 3A). Conversely, we have shown that Gac-mediated regulation occurs through the AmrZ repressor. This repressor has been shown to mediate the transition from motile *P. aeruginosa* cells to the non-motile (aflagellated) mucoid phenotype [47]. In *P. aeruginosa*, this transition occurs during chronic infections and the expression of *amrZ* requires the AlgU sigma factor. Similarly, in *P. fluorescens* F113 we have shown here a similar regulatory cascade. A functional *algU* gene is required for the expression of *amrZ* and repression of *fleQ*, resulting in reduced flagellar production.

**Figure 6:**
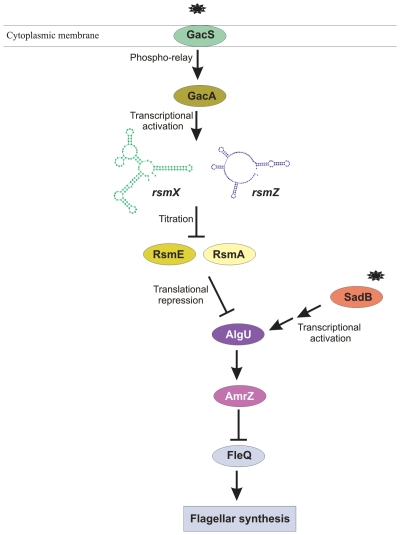
Hypothetical model for the environmental regulation of flagellar synthesis in *P. fluorescens* F113 through the Gac-SadB cascades. Arrows indicate positive control and perpendicular lines negative control.

We have also extended this regulatory network by showing that the expression of *algU* and possibly its translation, is co-ordinately regulated by the Gac system and the *sadB* gene. This has allowed us to propose a model for the environmental regulation of motility through repression of the synthesis of components of the flagellar apparatus (Fig. 6). According to this model, a yet unidentified environmental signal is perceived by the GacS protein that autophosphorilates and phosphorilates the GacA protein [49]. Phosphorilated GacA is responsible for the expression of small regulatory RNAs, able to titrate the Rsm proteins [5], [26]. The Rsm proteins are also able to bind a polycistronic mRNA, encoding the *algU*, *mucA*, *mucB* and *mucD* genes (Fig. 4) in competition with the *rsmX/Z* sRNAs, resulting in a decrease in the transcription/translation of the *algU* gene. The *algU* gene is also transcriptionally regulated by SadB in response to a cytoplasmatic signal, possibly c-diGMP [55]. AlgU is the sigma factor required for *amrZ* expression and AmrZ downregulates *fleQ* expression, resulting in a lower production of flagellar components, including the flagellin FliC. This model links signal perception by a membrane receptor (GacS) and a cytoplasmic receptor (SadB) with the production of the components of the flagellar apparatus activated by FleQ and identifies AlgU as an important node for the environmental regulation of motility. In this sense, the *kinB* gene which requires AlgU for expression [56]–[58], is also implicated in motility regulation in *P. fluorescens* F113, since a *kinB* mutant shows hypermotility [33]. It has been also shown that in *P. syringae*, AlgW a periplasmic protease that controls the levels of AlgU, is a key negative regulator of flagellin abundance [59].

Gac-mediated positive regulation typically occurs in the transition from exponential to stationary phase. In this sense, the Gac system has been defined as a global activator of secondary metabolism in stationary phase. Furthermore, a relation between the Gac system and RpoS, the stationary phase sigma factor, has been described [14], [60], [61]. Here, we present evidence showing that in *P. fluorescens* the Gac system is also active during exponential phase, being able to repress flagellar synthesis at this stage. This role is also supported by the finding of *gacS* expression being maximal at mid-exponential phase [62]. Therefore, we propose a second physiological role for the Gac system: the repression or downregulation of specific traits like flagella synthesis during exponential phase.

## Methods

### Bacterial strains, plasmids, and growth conditions

Bacterial strains, plasmids and primers used in this study are shown in Table S1 and S2. All the *Pseudomonas fluorescens* strains used here are derivatives of the biocontrol strain F113 [63]. All the PCR fragments obtained in this study were initially cloned in the pGEM®-T Easy vector according to manufacturer's instructions (Promega). Mutants were obtained by single homologous recombination of amplified internal fragment from the gene using primer pairs vfrF-vfrR, amrZF-amrZR and algUF-algUR (Table S2) and cloned into the suicide vector pVIK107 [64], pK19*mobsadB* [65] or pG18*mob*2 [66]. Mutants were checked by Southern blotting and by PCR. Overexpression of *rsmA*, *rsmE*, *rsmI*, *rsmX*, *rsmY*, *rsmZ* and *amrZ* genes was achieved by cloning them under the control of the IPTG-inducible promoter present in the pVLT31 plasmid [67], for this purpose primers rsmAextF-rsmAextR, rsmEextF-rsmEextR, rsmIextF-rsmIextR, rsmXextF-rsmXextR, rsmYextF-rsmYextR, rsmZextF-rsmZextR and amrZextF-amrZextR were used (Table S2). Hemagglutinin peptide (HA) was fused in-frame to RsmA protein at the C-terminal by PCR using primers rsmAextF and HArsmAR (Table S2), and the PCR product was ligated to pVLT31 vector. Plasmids were mobilized into *P. fluorescens* by triparental mating, using pRK600 as the helper plasmid [68]. *P. fluorescens* strains were grown in SA medium [69] overnight at 28°C; solid growth medium contained 1.5% (w/v) purified agar. *Escherichia coli* strains were grown overnight in Luria-Bertani (LB) medium [70] at 37°C. The following antibiotics were used, when required, at the indicated concentrations: rifampicin (Rif), 100 µg/mL; ampicillin (Amp), 100 µg/mL; tetracycline (Tet), 10 µg/mL for *E. coli* or 70 µg/mL for *P. fluorescens*; kanamycin (Km), 25 µg/mL for *E. coli* or 50 µg/mL for *P. fluorescens*; and gentamicin (Gm), 10 µg/mL for *E. coli* or 4 µg/mL for *P. fluorescens*.

### Transmission electron microscopy

Formvar-coated grids were placed on the top of a drop of bacterial culture either at 0.3 or 3.5 O.D._600_ for 30 s to allow bacterial adhesion. Liquid was eliminated with filter paper and grids were stained for 1 min with a 1% solution of potassium phosphotungstate and washed 3 times for 1 min with a drop of water. Grids were air-dried and observed in a Jeol JEM1010 microscope.

### DNA techniques

Standard methods [71] were used for DNA extraction, gene cloning, plasmid preparation and agarose gel electrophoresis. Southern blots were performed with a non-radioactive detection kit (DIG Luminescent Detection Kit for Nucleic Acids), and a chemiluminescence method was used to detect hybridization signals according to the instructions of the manufacturer (Roche Boehringer Mannheim). PCR reactions were performed using the *Tth* enzyme (Biotools) under standard conditions. DNA sequencing was done by chain-termination method using DyeDeoxy terminator cycle sequencing kit as described by the manufacturer (Applied Biosystems).

The sequences of the *P. fluorescens* F113 *rsmA*, *rsmE*, *rsmI*, *rsmX*, *rsmY*, *gacS*, *sadB*, *vfr*, *amrZ*, *algU-mucA-mucB* has been deposited in GenBank under accession numbers: *rsmA* EU165536, *rsmE* EU165537, *rsmI* JN382566, *rsmX* JN382569, *rsmY* JN382570, *gacS* JN382567, *sadB* JN382568, *vfr* JN382563, *amrZ* JN382562, *algU*-*mucA-mucB* JN382565. The complete genomic sequence of *Pseudomonas fluorescens* F113 has been deposited in GenBank under accession number CP003150

### Swimming assays

SA medium plates containing 0.3% purified agar were used to test swimming abilities. The cells from exponentially growing cultures were inoculated into the plates using a toothpick. Swimming haloes were measured after 18 h of inoculation. Every assay was performed three times with three replicates each time.

### Protein extraction and Western blots

Proteins were extracted from 200 mL exponential (O.D._600_ = 0.3) and stationary (O.D._600_ = 3.5) phase grown cultures. In order to detach the flagella, the cultures were agitated by vortexing for 2 min and then centrifuged for 20 min at 12,000 r.p.m and extracellular proteins were extracted from the supernatant by precipitation for 2 h at 4°C with 10% (w/v) trichloroacetic acid, followed by two washes with chilled acetone, and were finally resuspended in Laemmli buffer [72]. Proteins were resolved by 12% SDS-PAGE and stained with Coomassie blue. The same electrophoretic conditions were used for Western blotting. Acrylamide gels were transferred onto nitrocellulose membranes for 1 h under standard conditions. The membranes were incubated with a 1∶10,000 dilution of an anti-flagellin antiserum [40] for 16 h at 4°C and then with a peroxidase-tagged secondary antibody (anti-rabbit immunoglobulin) for 1 h at room temperature. The enhanced chemiluminescence (ECL) method and Hyperfilm ECL (Amersham Biosciences) were used for development.

### Gene expression analysis

Total RNA was extracted using Trizol® according to manufacturer's specifications (Invitrogen) from *P. fluorescens* strains grown at 0.8 O.D._600_ in LB medium. Genomic DNA remains were removed by RQ1 RNase-Free DNase treatment (Promega) for 30 minutes at 37°C. After that, RNA was purified using Trizol®. The concentration of RNA was spectrophotometrically determined in a Nanodrop® and integrity was verified in denaturing agarose gels. All RNA samples were stored at −80°C.

RT-PCRs were carried out using Illustra Ready-To-Go™ RT-PCR Beads kit from Amersham GE Healthcare. qRT-PCRs were performed in two steps: a first step of cDNA synthesis using the SuperScript®III First-Strand Synthesis System from Invitrogen and a second step of qPCR using the Power SYBR®Green PCR Master Mix from Applied Biosystems. In both cases, gene expression was measured into different backgrounds and normalized by using *16S* RNA as internal control. Every assay was performed three times with three replicates each time.

### RNA immunoprecipitation

RNA immunoprecipitation (RNA-IP) was performed using the same procedure as that described by Lin *et al.* [73] with some modifications. Briefly, 3 h post-induction with 1 mM IPTG, F113 strain harbouring the pVLT31-rsmAHA plasmid (Table S1) was fixed with 1% formaldehyde for 10 min at room temperature. Cross-linking was quenched by adding glycine to a final concentration of 120 mM, and then cells were sedimented by centrifugation at 5,000 rpm for 15 min at 4°C and washed twice with ice-cold Phosphate-buffered saline (PBS). The cells were lysed in a non-ionic sonication buffer (50 mM Tris-HCl pH 8, 150 mM NaCl, 5 mM EDTA, 1% Triton X-100, 0.5% NP-40, 1 mM DTT) containing protease inhibitor cocktail (Roche) and RNaseOUT™ (Invitrogen) and sonicated in a Bioruptor™ UCD-200 TM (conditions: power H, 30 sec ON-30 sec OFF, 10 min). Debris was removed by centrifugation, and lysate was divided and immunoprecipitated with 5 µg of either anti-HA antibody (12CA5, Roche) or appropriate control IgG (sc-2025, Santa Cruz Biotechnology) and 30 µL of Dynabeads® protein G (Invitrogen). After washing with sonication buffer four times and with TE twice at 4°C, samples were treated with RQ1 RNase-Free DNase (Promega) for 30 min at 37°C. Reverse transcription (RT) was carried out directly on magnetic bead-bound complexes with random hexanucleotide primers using SuperScript®III First-Strand Synthesis System (Invitrogen) according to the manufacturer's protocol. The cDNAs from pulled down fractions were quantified by qPCR as above using the primer pairs shown in Table S2. Every assay was performed three times with three replicates each time.

### Statistical methods

SPSS program was used for all statistical analyses. The data in Figs. 3A, 3C, 5A and 5B were compared using one way analysis of variance (ANOVA) followed by Bonferroni's multiple comparison test (set at 0.05) and in Fig. 2C using Student's t-test for independent samples (p<0.05).

## Supporting Information

Table S1 **Strains and Plasmids used.**
(PDF)

Table S2 **Primers used.**
(PDF)
