# Supplementary material for: The Gac-Rsm and SadB Signal Transduction Pathways Converge on AlgU to Downregulate Motility in Pseudomonas fluorescens
Source: PLoS One. 2012 Feb 20;7(2):e31765. doi: 10.1371/journal.pone.0031765 (PMC3282751; doi:10.1371/journal.pone.0031765)
Supplement: Table S1 — Strains and Plasmids used. (PDF) [file pone.0031765.s001.pdf]

Table S1. Strains and plasmids used

Strain or plasmid	Description	Reference or source
**Strains**		
E. coli		
DH5α	*E. coli* cloning strain	Gibco-BRL
P.fluorescens		
F113	*P. fluorescens* F113 wild-type, Rif^R	[63]
vfr ⁻	F113 *vfr* ⁻, Rif^R Km^R	This study
gacA ⁻	F113*gacA* ⁻, Rif^R Km^R	[32]
gacS ⁻	F113*gacS* ⁻, Rif^R Km^R	[32]
sadB ⁻	F113*sadB* ⁻, Rif^R Km^R	[33]
algU ⁻	F113*algU* ⁻, Rif^R Km^R	This study
amrZ ⁻	F113*amrZ* ⁻, Rif^R Km^R	This study
algU ⁻ *amrZ* ⁻	F113*algU* ⁻ *amrZ* ⁻, Rif^R Km^R Gm^R	This study
gacS ⁻*amrZ* ⁻	F113*gacS* ⁻*amrZ* ⁻, Rif^R Km^R Gm^R	This study
algU ⁻*sadB* ⁻	F113*algU* ⁻*sadB* ⁻, Rif^R Km^R Gm^R	This study
amrZ ⁻ *sadB* ⁻	F113*amrZ* ⁻ *sadB* ⁻, Rif^R Km^R Gm^R	This study
gacS ⁻ *sadB* ⁻	F113*gacS* ⁻ *sadB* ⁻, Rif^R Km^R Gm^R	[33]
**Plasmids**		
pVIK107	Suicide vector, Km^R	[64]
pK19*mobsacB*	pUC18 derivative *lacZ mob* site *sacB,* Km^R	[65]
pG18*mob*2	Suicide vector, Gm^R	[66]
pVLT31	IPTG-inducible expression vector , Tet^R	[67]
pRK600	Helper plasmid, Cm^R	[68]
pBG1529	*vfr* fragment in pVIK107, Km^R	This study
pBG1748	pVLT31 derivative containing the *rsmA* gene (p*rsmA*), Tet^R	This study
pBG1749	pVLT31 derivative containing the *rsmE* gene (p*rsmE*), Tet^R	This study
pBG1950	pVLT31 derivative containing the *rsmI* gene (p*rsmI*), Tet^R	This study
pBG1948	pVLT31 derivative containing the *rsmX* sRNA (p*rsmX*), Tet^R	This study
pBG1949	pVLT31 derivative containing the *rsmY* sRNA (p*rsmY*), Tet^R	This study
pBG1772	pVLT31 derivative containing the *rsmZ* sRNA (p*rsmZ*), Tet^R	This study
pBG1759	pVLT31 derivative containing the *amrZ* gene (p*amrZ*), Tet^R	This study
pBG1840	pVLT31 derivative containing the *rsmA-HA* fusion, Tet^R	This study
