# Supplementary material for: The Gac-Rsm and SadB Signal Transduction Pathways Converge on AlgU to Downregulate Motility in Pseudomonas fluorescens
Source: PLoS One. 2012 Feb 20;7(2):e31765. doi: 10.1371/journal.pone.0031765 (PMC3282751; doi:10.1371/journal.pone.0031765)
Supplement: Table S2 — Primers used. (PDF) [file pone.0031765.s002.pdf]

Table S2. Primers used

**Primer**	**Sequence5’→ 3’[a]**	**Purpose**
vfrF	ATGGTTGGTATTACCCCCA	To generate mutant
vfrR	GGCATCGGGTTGCTTGCA	To generate mutant
amrZF	GGGAACGCATTGCCGAAGTG	To generate mutant
amrZR	GCGTCATGGGCAATCAGCGAA	To generate mutant
algUF	TGCACGACACCCATGAAG	To generate mutant
algUR	GTCGACAAATCTTCGGGCA	To generate mutant
rsmAextF	ATGCTGATTCTGACTCGTC	To amplify complete gene
rsmAextR	TTAATGGCTTGGTTCTTCGT	To amplify complete gene
HArsmAR	TCCTGCAGTTAAGCATAATCTGGAACATCGTATGGATAATGGCTTGGTTCGTCG	To generate HA-tagged RsmA
rsmEextF	ATGCTGATACTCACCCGCAA	To amplify complete gene
rsmEextR	TCAGGGCGTTTGTGGCTTG	To amplify complete gene
rsmIextF	CGGGTGTCTTGAGAACAGGAA	To amplify complete gene
rsmIextR	AAGCACGACGTACCCAAGCA	To amplify complete gene
rsmXextF	AGATGGTAGCTATGTCGTC	To amplify complete gene
rsmXextR	AGCAGCAACGGCGTGTAAG	To amplify complete gene
rsmYextF	ACACATCGTGCTGGCGATT	To amplify complete gene
rsmYextR	GAAACGCAACCAGAAGCAGC	To amplify complete gene
amrZextF	ATGCGCCCATTGAAACAGG	To amplify complete gene
amrZextR	TCAGGTCGCGTCTGCGG	To amplify complete gene
rsmZextF	TAGCGTTACAGAGCAAGCCA	To amplify complete gene
rsmZextR	TTCGGATCAAGAAATCGCAG	To amplify complete gene
fleQF	GGCACCAGCCGGGCGAT	For *fleQ* expression
fleQR	TGACCTGCATCGGCAGCG	For *fleQ* expression
qfleQF	TGCTGATCCTGGGTGAGTC	For *fleQ* expression (qPCR)
qfleQR	ACTCGCTCTCCAGCAACTC	For *fleQ* expression (qPCR)
fliCF	AACTCCAGCCGTGCTGA	For *fliC* expression
fliCR	GCAGCTGGTTGGCCTGG	For *fliC* expression
qfliCF	TTCCGATGCTCTGTCCACTTC	For *fliC* expression (qPCR) and RNA-IP
qfliCR	GATACCGTCGTTGGCGTTCTT	For *fliC* expression (qPCR) and RNA-IP
16SF	TCAGTCACACTGGAACTGAG	For *16S* expression
16SR	CAGGCGGTCAACTTAATGCG	For *16S* expression
qamrZF	ATGCGCCCATTGAAACAGG	For *amrZ* expression (qPCR)
qamrZR	GAGAGTTGTCGGAAGCGTTGA	For *amrZ* expression (qPCR)
qalgUF	GCAGGATGTCGCTCAGGAAG	For *algU* expression (qPCR), RNA-IP and intergenic RT-PCR
qalgUR	AGTTTTTCGCCGTGTTGATGG	For *algU* expression (qPCR) and RNA-IP
qmucAF	GACAACGAAGCGGACGAAC	For RNA-IP and intergenic RT-PCR

qmucAR	AGGCGTGGAAGCAACAAGTC	For RNA-IP and intergenic RT-PCR
qmucBF	TATTCACGCTTCTGCTTGGTG	For RNA-IP
qmucBR	CTCTGCTGTTGCTCGGCTTGT	For RNA-IP and intergenic RT-PCR
qmucBF2	ACGGTGGTCGGTGAGATTCCT	For intergenic RT-PCR
qmucDF	CCTGATTTCACGCAACTGGTC	For RNA-IP
qmucDR	GCATCTGCTGGTCCGATACAC	For RNA-IP and intergenic RT-PCR
qhcnAF	TTCAGACCTTCTACGCCGAAA	For RNA-IP
qhcnAR	GGACTGGATGACGGTGAGGA	For RNA-IP

[a]HA sequence is underlined for HArsmAR primer
